# The (gradual) rise of memory inflation

**DOI:** 10.1111/imr.12653

**Published:** 2018-04-17

**Authors:** Paul Klenerman

**Affiliations:** ^1^ Peter Medawar Building for Pathogen Research and Translational Gastroenterology Unit University of Oxford Oxford UK

**Keywords:** adenovirus, CD8^+^ T cell, cytomegalovirus, exhaustion, inflation, memory, vaccination

## Abstract

Memory inflation, as a term, has been used for 15 years now to describe the longitudinal development of stable, expanded CD8^+^ T memory pools with a distinct phenotype and functional profile which emerge in specific infection and vaccine settings. These settings have in common the persistence of antigen, especially cytomegalovirus infection but also more recently adenoviral vector vaccination. However, in contrast to chronic infections which lead to “exhaustion” the repeated antigen encounters experienced by CD8^+^ T cells lead to development of a robust T‐cell population structure which maintains functionality and size. In this review, I will discuss how the ideas around this form of memory have evolved over time and some new models which can help explain how these populations are induced and sustained. These models are relevant to immunity against persistent viruses, to novel vaccine strategies and to concepts about aging.

## 1 INTRODUCTION

The term T‐cell memory “inflation” was first used 15 years ago to describe a phenomenon seen after infection of inbred mouse strains with murine cytomegalovirus.^1^ It is a striking phenomenon that was made quite evident by the use of MHC‐peptide tetramers to track responses in the infected animals over time.^2^, ^3^, ^4^ The term is now in reasonably common use^5^, ^6^ and the actual features of the responses induced which can be called “inflationary” are quite robustly reproducible between different laboratories using different virus stocks, mouse lines, and infection protocols. However, the immunologic phenomenon in the original papers was not tightly defined. This defensible as in some ways it was so distinct and obvious it did not appear to need a definition. Furthermore, a set of features relating to the scale and quality of the response seem to be inherently linked. Nevertheless, the original studies were describing 1 set of immune responses in the mouse cytomegalovirus system—so although these features overlap very substantially with others within that system and elsewhere, and they can all be described broadly as “inflationary”, each infection and species has its own specific version of immunity. Thus, some better definition would be useful in allowing comparisons between different settings and also in framing the interesting and important questions about what drives this kind of immune response.

The situation is quite similar to that seen earlier in relation to the term immune “exhaustion”, which was first coined by Rolf Zinkernagel's team studying features of persistent lymphocytic choriomeningitis virus (LCMV) infection.^7^ What was seen in those studies (performed in the days before tetramers were available, but making use of transgenic models to track responses), was a loss of T‐cell reactivity and ultimately physical deletion of antiviral responses. Again, the term is a clear and useful description of the phenomenon seen, although a spectrum of exhaustion phenotypes can be seen even within the same animal, which varies over time and between specificities. Following work by other groups, the phenomenon was redefined using a more graded system based on analysis of specific functions but it was not until the identification of specific markers linked to the gene expression profile of exhausted T cells discovered by the group of Rafi Ahmed over a decade later, that the phenomenon took on a more concrete mechanistic explanation.^8^ Even so, expression of single molecular markers such as PD‐1 is insufficient to really provide the pinpoint definition that would be ideal, and definitions based on gene expression or epigenetic regulation are somewhat complex.^9^ So, despite a huge amount of research effort (significantly more than has been expended on MCMV models) and a wealth of molecular data and elegant models, it is still actually quite hard to provide a robust and watertight definition of T‐cell exhaustion even 25 years after its discovery—even though broadly everybody knows what it means.

The analogy with exhaustion is not entirely academic as the conceptual framework and the molecular dissection of T‐cell exhaustion have been extraordinarily useful in the analyses of specific immune responses and in translational studies. Thus, even though exhaustion is quite variable and many aspects hard to define perfectly, interrupting key points in the system can reverse the phenomenon and lead to impressive in vivo impact. Also, since the phenomenon is reproduced across so many immunologic model systems, and parallels are easily seen in human immunity, it is perhaps not surprising that reversal of exhaustion through checkpoint blockade (eg, blocking of PD‐1 interactions) has a substantial impact in certain cancers.^10^ It is also interesting that the biggest effects have been seen in cancers, while many of the key original discoveries were made in virology.

So, with this perspective it should encourage a very optimistic approach to studies of memory inflation. In a lot of ways, it represents the “flip‐side” or “mirror‐image” of exhaustion in that prolonged exposure to a chronic virus infection enhances rather than inhibits immune reactivity. Like exhaustion, it is a clear phenomenon seen in many different models that is reproducible between mouse and man. And like exhaustion, there is no one simple marker or transcription factor which can be used to clearly mark cells—it relies on a combination of features. Although we do not have a perfect definition yet (and I attempt to improve a little on this state of affairs below), there is a good working agreement between groups where the features are well recognized. Where the analogies diverge at this point is we lack the molecular and genetic descriptions of inflation—not completely but at least to the same depth as for exhaustion—and we lack some of the critical conceptual framework describing its development and therefore the toolset to modulate it. However, the field is younger and smaller and we can learn a lot from those working on other aspects of T‐cell biology, so overall it seems quite possible that similar conceptual and also translational breakthroughs can be made.

In this review, I will discuss the current ideas about memory inflation and how they are derived and lead on to some of the open questions in the field that might be answerable soon. Firstly, I will discuss the origins of the idea and try and to develop a definition that is a little better than what is currently typically applied (which mainly relies on magnitude). Following this, I will discuss the phenotypes associated with inflationary cells and how these compare in different models. The next questions to address relate to the in vivo function of inflationary populations and the mechanisms which sustain them. I will explore the models which could explain the development of memory inflation across the different settings where it is found and some of the pros and cons of these explanations.

## 2 THE ORIGINS OF MEMORY INFLATION

The term memory inflation was born out of studies conducted by Urs Karrer and Sophie Sierro at Oxford using MHC Class I‐peptide tetramers to study CD8^+^ T‐cell responses against MCMV.^1^, ^2^, ^10^ At this point, the discovery of epitopes and virus‐specific T cells in the MCMV model was based on a good deal of careful work originating from the Koszinowski laboratory and peptide mapping by Matthias Reddehase in the BALB/c model, which had already described the specificity, the scale and some key aspects of the phenotypes of such populations.^11^, ^12^, ^13^ However, epitopes in the C57BL/6 model (ie, restricted by H‐2b as opposed to H‐2d) were not yet described. Using a recombinant virus expressing well‐described epitopes from influenza nucleoprotein (NP) and LCMV glycoprotein (GP) expressed at the C terminus of the immediate early 2 (IE2) molecule (which is redundant for full viral replication), C57BL/6 mice were infected and immune responses tracked over time.^2^ In fact, the initial results were rather disappointing, since acutely very little was seen. However, later on an accumulation of responses against the recombinant epitopes were observed which gradually increased over many weeks. Of note (and a further source of complexity in some studies), the immunodominant epitope in LCMV is a 9mer, KAVYNFATC, restricted by Db, and this is the common focus of most models. However, the inflationary response seen using the recombinant MCMV was directed against the alternative epitope which is an 8mer AVYNFATC restricted by Kb. The reason for this is still not fully defined, but could relate to competition or processing as discussed further below.

Studies using the same approach of longitudinal tracking by MHC‐peptide tetramers based on some of the previously described epitopes in the BALB/c system revealed similar profiles to the immune response, although since these were endogenous epitopes with a clear track record of detection (ie, relatively immunodominant), the populations were also observed during the early phases of infection.^1^, ^3^ Nevertheless, the apparent “memory” responses to some epitopes did not show classical contraction and indeed the phenomenon of late expansion was also seen.

Thus, the original term was born out of studies simply tracking responses in immune‐competent mice using the newly available tools and flow cytometry—and focusing on a numerical expansion which was observed at very late time‐points, well beyond the original control of the infection. It was also very striking that the later “inflation” was not dependent on the initial immunodominance of the response—this was especially obvious in the recombinant model.^2^ At a similar time to this, Ann Hill's group performed some very detailed mapping experiments using vectors and peptide libraries to map the dominant CMV‐specific CD8^+^ T‐cell responses in the C57BL/6 mouse. Thus in different mouse strains, an “inflationary” and a classical response were seen to evolve in parallel against distinct peptide epitopes even within the same animal.^3^, ^4^

Three other features of memory inflation also emerged from this early work with MHC‐peptide tetramers in standard immunocompetent mice infected with MCMV—and driven by analyses of human immune responses (which were completely transformed by this technology). These have also been broadly confirmed and extended by many groups over time and remain a relatively core set of inflationary features.^14^, ^15^ First was the distinct phenotype of the tetramer‐positive populations. These emerged as having “effector‐memory” profiles as judged by the expression pattern of molecules such as CCR7, CD62L, CD28, CD27, and CD127 (all low).^3^, ^16^, ^17^ This molecular pattern suggests homing to non‐lymphoid tissues and indeed the second feature is accumulation not only in blood and spleen where it was first noted but in many such tissues such as lung and liver, as opposed to lymph nodes.^1^, ^3^, ^13^ The third feature observed was maintained effector functions such as cytokine release—in contrast to exhausted CD8^+^ T cells.^8^ There are certainly some differences in the level of cytokine production comparing inflationary populations and classical non‐inflationary memory cells in the same model, but over time there does not appear to be clear attrition of such functionality (and the lack of transcriptional and phenotypic markers of exhaustion is consistent with this).^3^, ^4^, ^16^, ^17^ The findings were given some further relevance since they are quite parallel to those seen in human responses to HCMV—in other words very large responses identifiable by tetramer, with a phenotype described as “effector‐memory” (also CCR7, CD62L, CD28, CD27, CD127 low) and with maintained function. The accumulation of these with time is not so evident within an individual; although, they do appear to accumulate with age looking at populations cross‐sectionally. Thus, although they are by no means identical, the word “inflationary” has also been used to describe immune responses to HCMV, even though these are much more complex and diverse.^14^, ^18^, ^19^, ^20^ This is reasonable, given the very distinct features of both the mouse and human response, which overlap in many ways—but it depends on what is meant by memory “inflation” hence the attempt at a definition below.

## 3 WHAT IS MEMORY INFLATION NOW?

Since those early studies, which were restricted to MCMV, the idea of an inflationary memory response has been applied to other infection settings. In terms of mouse models, the most clear‐cut of these is the use of a replication‐defective recombinant adenovirus vector.^16^ At first sight, this ought not to work at all, since the features of the inflationary responses seen in MCMV seem to be closely linked to a persistent infection, with continuous generation of new peptides as the virus reactivates.^5^, ^21^ However, this model induces a set of immune responses which quite accurately reproduce many of the key features of memory inflation seen after MCMV infection. The responses to an expressed protein (driven typically by a CMV promotor, although this is not a requirement) such as beta‐galactosidase, can be tracked in the same way as an endogenous or recombinant MCMV antigen.^16^, ^22^ Over a range of doses, these show an increase and maintenance in frequency over time, a sustained effector‐memory phenotype and redistribution to tissues, accompanied by sustained functionality against 1 epitope (in the case of B‐gal, Kb‐restricted D8V). At the same time, a second epitope (I8V, restricted by Db) is co‐dominant after priming but undergoes classical memory evolution with the development of a contracted central memory pool. Further work on the model has indicated that the similarities are also seen at the underpinning transcriptional level.^16^, ^22^, ^23^, ^24^, ^25^

It is clearly very useful to have more than 1 virus/model that demonstrates memory inflation (as broadly defined) as it should allow a better triangulation of the key mechanisms which occur. It is also very interesting that the model should emerge from adenoviral vectors as these have special qualities which have been recognized by the vaccine community. Of all the recombinant vector approaches which have been used in an attempt to prime and expand human CD8^+^ T‐cell responses, adenoviral vectors are currently a leading technique.^26^, ^27^, ^28^ Thus, understanding some of the basic rules which allow induction of distinct long‐term memory responses could have direct impact on the development of vaccines—this is of course true not only for the adenoviral vectors but also for the CMV‐based approaches. However, adenovirus‐based strategies have the advantage that they are safe and well‐tolerated with a wealth of human early‐phase data in a range of vaccines (eg, Malaria, hepatitis C virus [HCV], Ebola, RSV).^29^, ^30^, ^31^, ^32^

However, how is it that a non‐replicative vector can elicit an immune response which shares so many features with a persistent herpesvirus? There is reasonable agreement that specific antigen re‐encounter is important in driving the inflationary phenotype over time. There is also evidence that adenoviral vectors are able to persist long term in tissues and continue to express antigen over this time.^16^, ^33^ Although measuring protein antigen directly is very hard the fact that it is present is most strikingly demonstrated by the responses of transferred T cells. In an experiment in which naive transgenic T cells specific for the D8V epitope from B‐gal were transferred into previously vaccinated mice, expansion and proliferation of these (originally naive) cells could be seen even if transfer occurred 100 days after the original inoculation.^16^ This is consistent with data from our group and others showing persistence of adenoviral DNA and also vector‐derived transcripts over such long periods.^34^ Single‐cycle MCMV vectors interestingly also give similar results.^35^

This persistence of antigen derived from a virus which is not classically persistently replicating can also drive memory inflation in human infections. Longitudinal studies akin to the mouse models have been somewhat hard to establish in CMV, but parvoviruses B19^36^, ^37^ and more recently PARV4^38^, ^39^ can be tracked following acute infection in adults and responses to specific epitopes show similar features of delayed expansion associated with maintained effector‐memory phenotypes and sustained functionality. Parvoviruses, such as these, do not undergo a latency programme, but can establish a long‐term DNA pool in tissues, and—if the T cells are to be trusted—these data indicate long‐term epitope production.

In a similar vein, responses following experimental adenoviral vector vaccination can be tracked in human volunteers (eg, following vaccination with the ChAd3 vector expressing hepatitis C virus NS3‐5B proteins). These do not show inflation in the original sense at all—responses overall show contraction in terms of frequency following priming (especially after boosting). However—they do show an interesting blend of memory populations, many of which have phenotypes and functional profiles closely aligned with those seen in human CMV‐specific populations, as well as those induced by MCMV and HuAd5 vectors in mice (for example as imaged using high content cytometry such as CyToF).^29^, ^40^ For example, they can show upregulation of CD57 along with a maintained and stable effector‐memory phenotype (CCR7 CD62L, CD28, CD27, and CD127 low).^16^

These features of CD8^+^ T‐cell responses have been observed in other infection settings such as chronic norovirus infection in mice,^41^ and some extreme responses to Epstein Barr Virus (EBV infection) in humans.^42^ Such responses, as well as those induced by adenoviral vectored vaccines in humans could be called “inflation‐like”. They are definitely non‐classical memory, but they vary widely in terms of frequency and, in many cases, lack any detail regarding distribution. Perhaps the key linking facet that could be used as the key to a definition is that they show features of antigen re‐encounter without any evidence of immune exhaustion. Thus, an updated definition of “inflationary” memory could include:

1. *Restricted contraction following priming, leading to a long‐term maintained memory pool*. There are certainly responses (including the pattern first identified by Anne Hill's group) which do show some contraction following priming, but plateau and are sustained at a level which is substantially greater than classical memory responses. (A good example is seen in the recent paper by Luka Cicin‐Sain using a recombinant MCMV expressing a—normally classical memory‐inducing—peptide from M45 in different contexts.^43^)
2. *A dominant and sustained “effector‐memory” phenotype* (this in itself is a spectrum of phenotypes^44^). It should be noted that not all antigen‐specific cells within an inflationary population possess this phenotype and indeed this structure may be an essential component of maintaining the population overall.
3. *A sustained effector functionality without features of immune exhaustion*. Features of immune exhaustion include surface marker expression (eg, PD1), in vitro functionality (eg, cytokine release), metabolic dysfunction, and disruption of transcriptional networks.^8^, ^9^, ^45^, ^46^

This encompasses the critical features of the phenomenon without being unduly restrictive. Ultimately, the best definition would be based on expression of transcription factors—although not perfect themselves these have great utility in the definition of regulatory T cells (FoxP3) and Th17 cells (RORgT)^47^ and also in T‐cell exhaustion (Eomes and T‐bet).^48^ Indeed, networks of transcription factors can be observed to be strongly co‐regulated in inflationary populations, although whether such a definition is robust across the models and has real utility is yet to be proven.^16^

**Figure 1:**
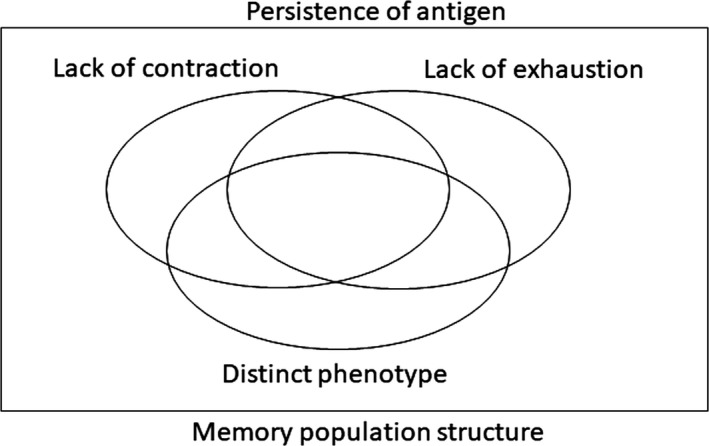
A pictorial “definition” of memory inflation. The definition includes the framing of the phenomenon within the context of antigen persistence and also includes the fact that not all T cells included in the response to a given epitope will have an “inflationary” phenotype. The 2 negatives in the definition (lack of full contraction and lack of exhaustion) could still be improved if a transcriptional programme can be defined more accurately which underpins the distinct phenotypic and functional profile seen

It should also be recognized that memory “inflation” is really referring to a dynamic immunologic phenomenon, which has many component parts—like immune memory itself. An “inflationary” cell is the hallmark of this process but in order to sustain inflation it seems very likely that a population structure which includes more conventional memory pools is required. Thus, the definition should acknowledge the diversity of memory states required to maintain inflation, as well as the striking populations themselves and the persistence of the antigen to which they are responding (Figure 1).

Overall, with the data which have emerged from different models pointing in the same direction, and with a definition with is broad enough to encompass the variations on the theme, while strict enough to have some rigor, it seems reasonable to conclude that memory “inflation” is a reproducible and well‐defined pathway of T‐cell differentiation in response to a subset of immunologic challenges. This is important as otherwise the discussion and focus is limited to cytomegaloviruses. These viruses manipulate the immune response in a particular way and set up lifelong persistence leading in some cases to very exaggerated effects. However, the actual pathway of differentiation appears to be much more widespread and the common features of this differentiation (and the pathways that underpin this) are probably relevant in many situations where it has so far not been explored.

### 3.1 Phenotypes of memory inflation

Since the populations of inflationary cells are large, these lend themselves to analysis by flow cytometry and more recently gene expression studies, such that many groups have described their distinct phenotypic characteristics on the most abundant cells.^17^, ^49^ Some of these have been discussed already as they are embedded in the definition—an effector‐memory phenotype as defined by lack of expression of lymph node homing markers such as CCR7 and CD62L.^3^, ^13^ It is certainly also the case that cells found in lymph nodes with these specificities retain expression of or re‐express such molecules, although these are a minority (in proportion).^50^

Other features which were recognized early on and reflect the patterns seen in human CD8^+^ T cell responses elicited by CMV include downregulation of the co‐receptors CD28, CD27 and also the IL7 receptor (CD127).^51^, ^52^ Again, those cells which retain a central memory phenotype and are found in lymph nodes have not undergone this downregulation (or have upregulated them following priming). Obviously, the interpretation of this is that such cells have lost the requirement for additional co‐stimulation and some cytokine support, (although IL‐2, for example, is certainly required).^53^ Consistent with this, genetic deletion of CD28 has a major impact on classical memory responses following MCMV infection, but a more limited effect on memory inflation over time.^51^ Some form of co‐stimulation (eg, via CD27)^54^ may be needed and there are some data that context‐dependent signaling through 41BB and OX40 is required for optimal generation of inflation in the long term.^55^, ^56^, ^57^ It is important to recognize the dynamic nature of some these effects—in most model systems inflation is best studied after 50‐100 days—while priming occurs in the first week (in the case of MCMV) or up to 3 weeks (in the case of adenovirus vaccination). There are data that suggest an important role for T‐cell help in generating optimal memory populations during priming and this includes an impact on some (but not all) inflationary populations.^58^ Interestingly in the adenovirus model, a lack of help leads to induction of exhausted responses following vaccination.^59^ The detailed function of this help has not been fully defined but it is possible that some of the impacts seen with disruption of signaling and cytokine pathways are at least partially via disruption of critical helper responses.^56^

These phenotypes have been well recognized and explored, but other surface marker and transcriptional changes are of equal interest. One such molecule is CX3CR1, which is sustained on inflationary populations found in blood and spleen, but expression is lost over time in responses non‐inflationary epitopes (ie, classical memory).^16^ CX3CR1 binds fractalkine, a chemokine important in tissue homing. It has recently been shown that CD8^+^ T cells expressing high surface levels of CX3CR1 lack proliferative capacity and have been described as “terminally differentiated”.^60^ To that extent, this fits with many descriptions of CD8 T‐cell phenotypes associated with CMV in mouse and man. Additionally, there exist subpopulations of cells that express intermediate levels of CX3CR1.^60^ These are of particular interest in the setting of inflation as they possess both an “effector‐memory” phenotype typical of sustained antigen re‐encounter, but also proliferative capacity. Furthermore, upon adoptive transfer to naive mice, such intermediate cells show the capacity to revert to a CX3CR1 low state more typical of the non‐inflationary pool. These experiments were originally performed in LCMV models, where memory inflation is not seen, but have important implications for MCMV and adenoviral models. A population of CX3CR1 intermediate cells that is sustained by antigen re‐encounter following priming could allow support of memory populations in blood and tissues which are not only differentiated but also sustained over time without contraction.

It is quite likely, judging by other markers, that CX3CR1 expression is tracked by several other molecules associated with the expression of the underlying transcription factors.^16^ So, the expression of an “intermediate” differentiation state which is linked to sustained proliferative capacity as well as the potential to further differentiate or revert is of some interest in models of memory inflation as discussed below. It may therefore be that CX3CR1 itself is of less interest in terms of a non‐redundant or critical function for the cells and more as a window onto an intermediate cell state which is less obviously seen with other cell surface markers. However, it is entirely consistent with data on human T cells which emerged from early CyToF studies showing the differentiation between central and effector‐memory pools, (including so‐called terminally differentiated populations) is a continuum.^40^, ^44^

Expression of classical inhibitory receptors (ie, as associated with T‐cell exhaustion) is not a feature of inflationary populations (although they may express PD‐1 during acute activation like all T cells, along with CD160, Lag3, BTLA, and Tim3).^16^ Also—as described above—loss of classical co‐stimulatory molecules is seen. However, other co‐inhibitory and co‐stimulatory receptor molecules are upregulated at the cell surface compared to conventional memory cells.^61^ These over‐expressed genes and molecules include *Klrc1* (NKG2A), *Klrg1* (KLRG1), *Klre1*,* Klra1* (Ly‐49c), and *Klrk1* (NKG2D).^16^, ^17^ The functions in vivo are not yet well explored but it might be they perform some similar “tuning” activity eg, in preventing overstimulation and promoting long‐term survival. Expression of KLRG1—which has well‐documented inhibitory functions—is often used as a marker for this type of cell population.^60^, ^62^ In contrast, NKG2D is stimulatory, so the balance between signaling through such receptors may be critical for activation as it is in NK cells.

As already mentioned, the surface phenotypes seen (which also include upregulation of effector molecules such as granzymes and perforin) allow easy recognition of the cells and provide some clues as to their function and regulation—but this widespread set of changes is driven ultimately by a smaller network of transcription factors. This is clear from first principles but also can be seen by principal components analysis of gene expression profiles from inflationary and non‐inflationary populations over time.^16^, ^49^ As would be expected, very large numbers of genes are differentially regulated between M38 (inflationary) and M45 (non‐inflationary) CD8^+^ T‐cell populations at late time‐points (over 1000 upregulated and 500 downregulated). But the populations can be equally well segregated using a smaller set of designated transcription factors.

One hope would be that there would be underlying all of this a “master” transcription factor that drives memory inflation. This is perhaps unlikely as in terms of functions there is no unique function or indeed phenotypic marker that completely defines the population (unlike say, IL‐17 secretion). However, sustained expression of TBX21 (T‐bet) is a clear finding of such analyses. T‐bet is another example (like CX3CR1) of a gene which is highly expressed early on in both inflationary and non‐inflationary pools, but which diverges over time, with maintenance in the inflationary populations and lower expression levels in the classical memory cells.^16^, ^49^ Inflationary cells derived from different models and species tend to show high levels of T‐bet with relatively low levels of Eomes (the opposite situation from immune exhaustion).^16^, ^40^ In exhaustion, the gene networks associated with T‐bet expression are also found to be disrupted compared to functional memory,^46^ while in memory inflation these remain intact (manuscript in submission). T‐bet has a very well‐defined role in driving effector CD8 T‐cell responses.^48^ Thus, a functional and sustained T‐bet‐driven gene network has a claim on a core transcriptional feature of memory inflation, but some further work is needed to establish if this is cause or correlate.

One feature of memory inflation which has not been so well explored but could be relevant to the overall phenotype of the cells is the nature of their metabolic regulation and the balance between different energy sources. In immune exhaustion, severe dysregulation of mitochondrial function is observed which may have impact on cellular functions and ultimately survival.^45^ Inflationary cells must establish some form of long‐term balance between glycolysis and fatty acid metabolism (oxidative phosphorylation) which allows long‐term survival but “effector” type functionality. Currently, we lack data which specifically address the development of metabolic phenotypes associated with memory inflation in the established murine models, although genes associated with metabolic pathways feature prominently in the transcriptional analyses.^16^, ^49^ Relevant data from human studies using effector‐memory pools reveal a metabolically stable pool of cells with a capacity to rapidly upregulate glycolysis upon restimulation.^63^ Studies of such cells in murine models will be of interest, although since there are important differences between human effector‐memory cells which express CD45R0 or CD45RA (TEM vs TEMRA populations) defining the exact equivalent in mice of a CD45RA+ revertant memory cell still warrants further work.

Related to the fundamental cell biology of memory is an observation that autophagy is required for the development of memory inflation.^64^ This finding is not unique to inflation as T cell‐specific deletion of key autophagy gene ATG7 affected responses not only to MCMV but also to influenza. Nevertheless, the dynamics of this response in the autophagy‐deficient mice is quite striking since a normal M38‐specific population was induced at priming, but the population subsequently collapsed and could not undergo further expansion. The implication of this study was that accumulation of defective mitochondria or other toxic cellular products such as reactive oxygen species or lipids leads to lack of evolution of cells into the memory phase, although the exact mechanisms still need to be further established. Autophagy‐deficient cells showed poor mitochondrial health and increased cell death, associated with upregulation of glycolysis pathways—they also responded poorly to proliferative stimuli. The speed of the collapse of population suggests that at early phases of infection the M38‐specific cells are dominated by short‐lived pools. The lack of repopulation later on may reflect the critical requirement for ongoing proliferation required to maintain the inflationary population—this is potentially impacted upon inflationary populations by aging.^65^

Overall, these data on phenotype as they have evolved over time suggest an interesting balance has been struck in the inflationary cells between a lifestyle as an effector cell and that of a memory cell for long‐term survival. Many of the transcriptionally regulated genes (up and down) reflect not overt immunologic functions but the basics of cell biology—notably cell cycle genes, modulators of survival and apoptosis, and metabolic regulation. Further studies to explore at what stage these programmes become embedded in the cell population and to what extent they are indeed fixed properties of the cell or rather transient could be valuable—they not only help cement the definition of the cell population of interest but also offer opportunities for modulation in the context of vaccination.

### 3.2 In vivo functions of inflationary populations

There are 2 elements to the question of functionality of inflationary populations—firstly what functions can they perform eg, in terms of cytokine production, killing, proliferation etc. and secondly what is their actual role in vivo. The latter question is obviously most relevant in the case of MCMV. Nevertheless, since in the case of adenovirus vector vaccination the CD8^+^ T‐cell response is not playing any role at all in the control of the virus, given it is non‐replicative, it is clear that inflationary populations do not necessarily play an equal role in vivo in all settings.

In the case of MCMV, it seems very plausible that—since on the one hand inflationary T cells show evidence of recurrent encounter with antigen and on the other CD8^+^ T cells are known to play a role in control of viral recrudescence long term—the specific responses seen to inflate are involved in viral control. (This is a little different from showing a role in viral control acutely or in the context of adoptive transfer during immunosuppression). One piece of evidence that the inflationary population is required to maintain latency comes from studies by the Reddehase group of a key early‐expressed epitope in IE1 (dominant in the BALB/c model).^5^, ^21^, ^66^ It was previously noted that IE1 transcripts can be detected eg, in the lung, but IE3 was not found, suggesting a checkpoint restricting progression. IE1‐mutated viruses showed a distinct pattern of transcription such that RNA transcripts could continue to be produced from IE1, until a new checkpoint was reached in the replication process. These data point to a specific role in ongoing surveillance, although even the IE1‐mutated virus did not reactivate. From the phenotypic perspective, it is not so obvious that non‐inflationary responses (such as M45‐specific cells in the B6 model) are still re‐encountering antigen although it may be still the case that this does occur in specific tissues and without the same clear‐cut changes seen in circulating tetramer+ populations.

Another interesting observation which sheds light on the potential role of inflationary populations in the MCMV model is the “bounce‐back” seen after transient depletion. Peptide‐specific cells can be depleted in vivo using saporin‐labeled tetramers.^23^ The depletion is very effective but in the case of M38‐specific populations specific for MCMV a very rapid re‐expansion was seen once therapy was stopped. The net result of the transient depletion was the emergence of a stable population at a higher “set‐point”—as high as 40% of the total CD8^+^ T‐cell population. Interestingly, although no virus reactivation was measured, there was evidence of proliferation of (non‐inflationary) M45‐specific cells over this period, which could indicate some transient loss of viral control. Depletion of M45‐specific cells had no impact on M38‐specific populations, but M45‐specific populations also showed bounce‐back to the original set‐point of memory without overshoot.

Another curious feature of this depletion experiment^23^ was the maintenance of the phenotype of the inflationary population—very high levels of Ki‐67 were observed during the recovery period, with evidence of activation—but throughout this period the “effector‐memory” or “highly differentiated” phenotype described above was maintained (including expression of NK‐associated receptors). There are 2 likely explanations for this—one is that these proliferating cells are derived from a central memory pool which can expand, differentiate, and redistribute to organs very rapidly.^50^ The other is that an “intermediate” pool of cells exist (as described for the CX3CR1‐intermediate pool) which possesses features of differentiation but retains proliferative capacity.^60^ Since these “intermediate” cells may be found in organs, this would allow for some local expansion in situ if antigen is presented.^67^

A further recent piece of data which might be relevant to this expansion potential and malleability was derived from a study where inflationary populations were depleted by superinfection.^25^ In this case, inflationary responses directed against B‐gal D8V in the adenovirus vector model were shown to be depleted markedly by later superinfection by MCMV (but not if given together or if the inoculation order was reversed). This depletion of inflationary responses occurred acutely, before expansion of MCMV‐specific responses, and appears to be mediated via Fas‐dependent killing. Here a bounce‐back is not seen (possibly since the adenovirus vector is non‐replicative and not actively suppressed by the CD8^+^ T cells induced), and a stable inflated memory response can be observed long term. This response can be further rapidly re‐expanded in vivo, however, by re‐exposure to the same adenoviral vector. The cell phenotype is once again tightly maintained. Data with similar implications are obtained by experiments in the MCMV model, where the populations expanded by low‐dose infection can be further boosted (and driven toward a more differentiated phenotype) in response to reinfections.^49^, ^68^, ^69^

All these data indicate that the function of the inflationary populations is quite dynamic in vivo and likely to be influenced continually by stimulatory and inhibitory stimuli. In fact, it is remarkable how stable the populations appear to be given the potential for substantial flux through death and proliferation. Likely, the best way to interrogate the in vivo function and regulation of inflationary populations in a virus infection is to specifically disrupt them once established—although the overlapping functions of many populations with different specificities, and the lack of a unique marker for inflationary cells might make this experimental approach difficult. Furthermore, MCMV itself is such a highly adapted pathogen with a range of immune evasion genes at its disposal that the host virus interplay is very complex and single interventions may be of limited impact.

An interesting alternative model which includes a live virus in which to explore this is murine norovirus. Although this is typically considered an acute infection, chronic infections can also be established with specific viral strains. In one of these, a persistence of virus is associated with emergence of an “inflationary” as opposed to an exhausted CD8^+^ T‐cell phenotype in the gut.^41^ It is not clear why the virus is able to trigger long‐term functional T‐cell expansion like CMV, without the same virologic strategy, but fundamentally some mismatch between where virus is presented to the immune system and the cells in which it is successfully replicating is postulated (ie, T cell “ignorance”). Thus, the CD8^+^ T cells appear to see just enough antigen to maintain the size and differentiation state of the population without providing full protection against replication.

**Figure 2:**
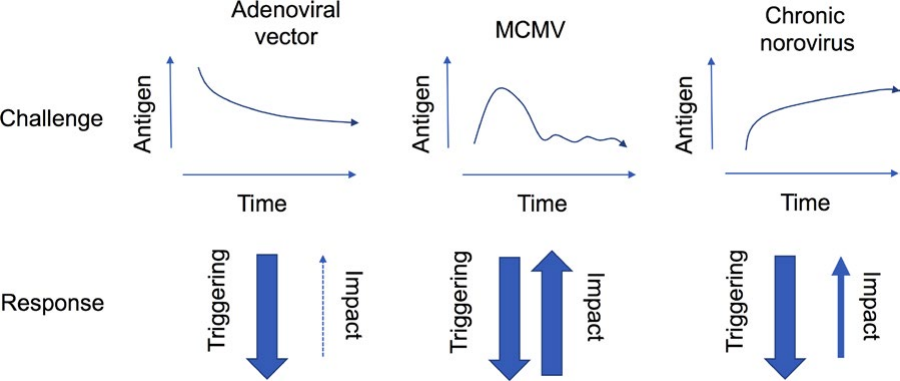
Memory inflation and viral control. The infections discussed represent different examples on a continuum of viral replication dynamics. The inflationary response can vary between an essential role in virus suppression to effectively a bystander role

Overall, putting together data from adenovirus vectors, through norovirus to MCMV, a spectrum of in vivo functions can be observed (Figure 2). In each case, we can detect a set of expanded T cells with maintained capacity for cytokine release, killing and proliferation. In the case of adenovirus, this is effectively irrelevant to the control of the vector which is replication‐deficient. With MCMV, it seems likely that each inflationary population is playing its part in control of the virus through some antiviral activity in the face of continuous reactivation. Norovirus presents an alternative outcome, where CD8^+^ activity can play a role in acute viral control, but the virus is able to establish an effective niche for survival with high levels of replication.

## 4 MECHANISMS UNDERLYING MEMORY INFLATION

### 4.1 Antigen presentation

One of the most obvious questions about memory inflation is why some epitopes and not others? In mouse models where this can be examined, there are a number of responses which are induced acutely but only some lead to inflation.^4^, ^70^ The first point is that it depends on the viral context, since epitopes which show inflation in the adenovirus or MCMV models do not induce inflation if presented in other recombinant settings such as vaccinia viruses—in other words it is not an inherent property of the peptide (for example related to some aspect of cross‐reactivity).^16^, ^22^ Within a given model where inflation can occur, the answer does not appear to be related to initial immunodominance, since even very subdominant responses can inflate at later time‐points.^2^, ^3^, ^4^, ^71^ Similarly, it is not dependent on the affinity of the epitope itself for MHC, since responses to the same epitope can be converted from a non‐inflationary to an inflationary type by modulating the epitope context.^2^, ^3^, ^71^

If not the peptide, then perhaps it could relate to the antigen. This is quite relevant in the MCMV model, where specific antigens are generated at different points in the replication cycle, and where in the BALB/c model, the first dominant inflationary response seen was to IE1, which is in the Immediate Early locus.^1^, ^13^ However, inflationary responses are seen against a range of epitopes from different proteins, and there are examples of proteins which include an inflationary and non‐inflationary response.^4^ Furthermore, in the adenovirus model, where there is 1 promotor and 1 protein transcribed, there is still the capacity to produce inflationary and non‐inflationary responses in parallel.^16^, ^25^ So, assuming the same rules apply, even if the level of protein antigen production could influence the abundance of a given peptide epitope derived from it, an additional selection step must occur to distinguish inflation‐inducing from non‐inflationary peptides.

The most evident explanation to date is that inflationary epitopes have distinct processing requirements. There are a few pieces of data which support this. Firstly, inflationary responses appear to target epitopes which are generated independent of LMP7—ie, immunoproteasome‐independent.^72^ Immunoproteasome dependence is not possible to predict exactly from sequence alone but functional experiments show consistent differences between inflationary responses in both the MCMV and the adenovirus models.^16^ Further, removing the requirement for processing by modulating the context of the epitope can have impact on memory inflation, particularly at the C terminus.^22^ This is easily performed in the adenovirus model where minigene vectors can be readily generated. It is also nicely shown in the MCMV model by making recombinant viruses where the immunodominant M45 peptide is relocated to the C terminus of M45.^43^ In this case, there is inflation demonstrated against the same M45 epitope expressed in the same open reading frame. This newly relocated epitope shows immunoproteasome independence. A related observation was made with a second epitope in the same location, where by adding alanine molecules at the N terminus of the peptide to improve processing also yielded memory inflation.^43^

Overall, the data so far indicate that efficient processing by a conventional proteasome is a key filter to provide a subset of peptides for memory inflation. There remain still other questions about peptide affinity and potentially also competition which can nevertheless influence either whether inflation occurs within this much smaller pool, or possibly the kinetics or hierarchy observed. One interesting observation relating to this comes from the use of a recombinant MCMV expressing the OVA‐derived and highly immunodominant peptide SIINFEKL (at the C terminus of GFP).^73^ In this infection model, the responses to conventional inflationary epitopes are suppressed and do not show phenotypic features of repetitive antigenic encounter. If co‐infection between the SIINFEKL recombinant and a wildtype MCMV occurs, then all responses show inflation. These data indicate that competition may prevent exposure of epitopes when co‐presented on the same cell.

In these experiments, it appears that SIINFEKL has a substantial kinetic advantage over the other peptides as well as a higher binding affinity for the same MHC molecule (Kb), so it perhaps represents 1 end of the spectrum, but it does prove the point that other filters exist which can have impact on which peptide‐specific responses dominate the inflationary pool. In experiments looking at competition in other contexts, the results are similar although less dramatic. For example, in a C57BL6 x BALB/c F1 mouse, there are potential responses to inflationary MCMV epitopes through H‐2d and H‐2b.^25^ Overall inflationary responses were seen but the responses to the BALB/c epitopes were not influenced by the presence of the H‐2b alleles, while there was a reduction seen in the response to for example M38 (which is a dominant epitope in the C57BL/6 model). Nevertheless, if MCMV and an adenoviral vector are co‐administered to a C57BL/6 mouse, then memory inflation can occur against multiple epitopes simultaneously—a result reflecting co‐administration of multiple adenoviral vectors^25^ and the MCMV co‐infection result described above.^25^

### 4.2 The nature of the antigen‐presenting cells

A common feature of the work on peptide selection for inflation has been the observation that there is a discrepancy between those epitopes which are presented during priming and those which are apparently presented over the long term. One explanation might be that they are presented on different cells. This is backed up by the observation that they have different processing requirements. Early studies in MCMV using viral mutants which lacked immune‐evasins showed—unexpectedly—little impact of such deletion on priming of antiviral responses and it was suggested that cross‐presentation might be an important mechanism for presentation during priming.^74^ The idea that an alternative (ie, non‐professional) presenting cell is critical for long‐term antigen presentation has attracted attention as a way of rationalizing the observed responses.

One set of experiments which yield data consistent with this was performed by the Oxenius group using MCMV infection of BATF3‐deficient mice.^75^ These mice lack dendritic cells critical for cross‐presentation and as predicted showed a major defect in priming across a wide range of epitopes. However, they show normal inflationary responses to M38 and m139 epitopes (the subdominant IE3 epitope showed an intermediate phenotype). Further work to explore this phenomenon using bone marrow chimeric mice has been performed with similar conclusions.^43^, ^50^, ^76^ In mice where cross‐presentation is blocked by reconstitution of the bone marrow with cells lacking the relevant MHC or TAP, priming is impaired but inflation is not (and vice versa). Thus, the presenting cell involved in inflation is not bone marrow‐derived.

Similar data come from the adenovirus model system. Here, work has been done using a recombinant virus expressing SIINFEKL, and bone marrow chimeras provide similar data (ie, bone marrow‐derived cells are important for priming but not for long‐term memory maintenance).^33^ Here, because the infection can be administered in a local fashion, it is also possible to remove lymph nodes and show an effect of such surgery on priming but not on long‐term memory (not described as inflationary in these studies, but showing comparable phenotypic features^34^, ^77^, ^78^). In a combination of such approaches, the authors conclude that both hemopoetic and non‐hemopoetic cell populations are required for optimal generation of memory (ie, functionally, numerically and with protective capacity).

**Figure 3:**
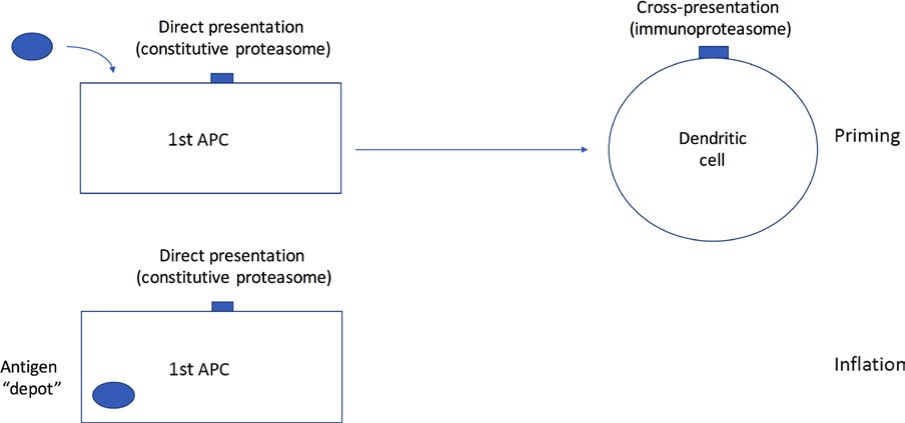
Antigen presentation and memory inflation. The infection/transduction of a stable cell type which is not a dendritic cell is suggested by different experimental data in MCMV and adenoviral models. Priming requires cross‐presentation and leads to generation of multiple responses to antigens which are not expanded during the memory phase—only those antigens presented on the unconventional APC drive memory inflation. Whether priming can additionally occur on the original APC is not fully defined, but is likely to be much less efficient

It is interesting that the adenovirus and MCMV models show experimental convergence here with quite overlapping findings regarding a critical importance of non‐hemopoetic or non‐professional antigen‐presenting cells in long‐term memory inflation. It is less evident whether non‐hemopoeitic cells can be really sufficient for priming although clearly for maximal early priming cross‐presenting DCs are involved^79^ and these cells can additionally prime the conventional (non‐inflationary) memory populations in parallel. Some debate still exists as to the nature of the non‐hemopoetic cell required. An obvious site is the lymph node since this is where the cells with high proliferative capacity exist (ie, those with a central memory phenotype) and there is evidence for their reactivation (upregulation of CD69, notably in mesenteric nodes in MCMV models).^1^, ^3^, ^50^ However, other data (including the adenovirus model accompanied by surgery) suggest alternatives may also exist.^33^ In the MCMV model, treatment with FTY720 which interferes with migration from lymph nodes did not affect memory inflation and it was suggested that exposure of blood‐borne cells to antigen on endothelial cells could provide the relevant source for maintenance.^67^ If this is the case, then proliferation would need to occur in an already partially differentiated cell population. It is quite possible that multiple depots of long‐term antigen exist and these may well vary between models—even of MCMV. Furthermore, given the lessons learned from the transient depletion experiments,^23^ disturbing the equilibrium of the finely tuned system can lead to rapid compensatory strategies. Combining data from both MCMV and adenoviral vector models, likely we are looking for depot cells which are very long‐lived, non‐hemopoetic in origin, lack immunoproteasomes at rest and provide ongoing stable antigen presentation (Figure 3).

## 5 MODELS FOR MEMORY INFLATION

A model for memory inflation should account for its apparent stability, but also the underlying dynamic nature of the populations which sustain it. Adoptive transfer experiments and other data suggest a half‐life of the cells of around 2 months.^17^, ^67^ Thus, although the actual fraction of cells proliferating on any 1 day is not high as caught in cross‐section (eg, via BrDU or Ki67 staining),^3^ there is nevertheless substantial turnover of the memory population. If it is turning over in this way based on proliferation of the cells which retain maximal potential, how is the “differentiated memory” phenotype and distribution maintained?

The simplest models from the start have worked on the assumption that the central memory pools of populations which are nevertheless dominated by inflationary phenotypes and behavior provide a core base for future maintenance and/or expansion of the overall population. This makes sense as this aspect of long‐term memory is very long‐lived and antigen‐independent. Such populations are enriched in lymph nodes and this is a classical site for restimulation where the conditions are optimal. A model should account for proliferation and additionally for the inclusion of such pools in the process.

**Figure 4:**
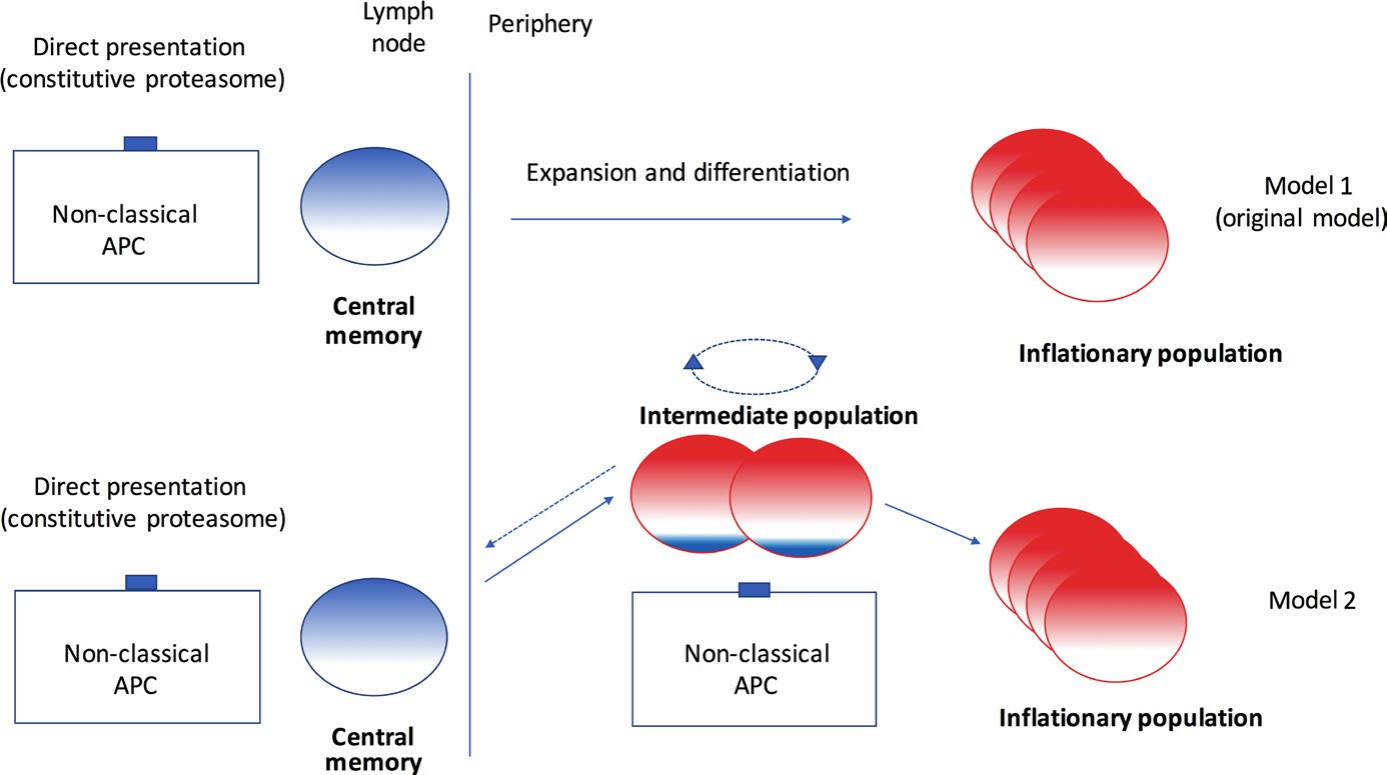
Models for memory inflation. The original model is based on 2 populations of cells, 1 in lymph nodes and the rest expanded in the periphery. Model 2 includes an intermediate cell type which does not possess lymph node homing markers but retains proliferative capacity. Exposure to antigen on a non‐classical APC in the periphery (at sites to be defined fully in the MCMV and adenovirus models) could drive proliferation and differentiation of these cells. They may also self‐renew or potentially de‐differentiate toward the long‐lived central memory pool

This may be sufficient and such a model, based on antigen re‐encounter in the lymph node and proliferation of retained central memory pools is definitely the simplest (Model 1 in Figure 4). However, it does not necessarily account for all the data, including the observation that blockade of lymph node egress does not have the expected impact on inflation.^67^ Further, the cells seen cycling immediately after tetramer‐based depletion already had a differentiated phenotype which may be explained by rapid differentiation but could reflect proliferation from an already partially differentiated pool.^23^

I would propose a new version of this model based on the idea that “intermediate” populations exist. The simplest version of this is the analysis of CX3CR1 mid cells, which are partially differentiated but retain proliferative capacity.^60^ This is a straightforward and classical way of dividing cell types on the basis of a single surface marker, but the evidence for intermediate cell types is much more profound than this.^80^ The examination of human CMV‐specific responses by phenotype (and additionally adenoviral vector‐driven responses) using high content cytometry methods such as CyToF reveals a spectrum of phenotypes.^40^, ^44^ So, the search for an intermediate cell type is in one sense very simple as we can be sure that cells developing along this spectrum pass through such states, although may be more complex than the use of a single marker. Nevertheless, the identification of such CX3CR1‐mid memory cells enriched in inflationary populations gives substantial impetus to this model (Gordon et al, manuscript in press).

Thus, in model 2 (Figure 4) there is (at least), 1 additional step in the pipeline from central memory to differentiated, expanded, and redistributed inflationary memory which occurs outside lymphoid tissues once the cells have egressed. This could occur in a single or multiple tissues and—as for the lymphoid presentation—the key cell type performing the antigen presentation needs to be identified. There is some intersection here with the discussion about the identity of the non‐professional antigen cell above. It seems likely that the development of the conventional memory pools and the central memory pools which feed the inflationary populations long term could both be provided predominantly through cross‐priming on dendritic cells. This would fit with the lymph node model—and long‐term depots of antigen in lymph nodes could sustain this. Distributed antigen in long‐lived tissue‐associated antigen‐presenting cells such as stromal and endothelial cells could have a role in phase 2 through engagement with the intermediate cell population.

The last part of the pipeline has 3 outcomes, each with some question marks over it. If the intermediate cell population in the periphery differentiates further, ultimately these pools of cells cannot revert, lose proliferative capacity and ultimately will die—presumably with a life span dominating the observed half‐life discussed above. This is the most clear‐cut route. Some of the proliferating population may also maintain themselves in an intermediate state—ie, allowing that part of the pool to survive longer and enhancing the overall expansion. Further, it has been shown that CX3CR1 mid cells can de‐differentiate over time and these could further reseed the central memory pool—likely the majority part which is mainly found in lymphoid tissue.^60^ Likewise, CD45RA+ T cells in humans can “de‐differentiate” and proliferate despite being commonly labeled as terminally differentiated.^81^, ^82^

This model (2) has some advantages over the original as it accounts for the activities of newly described populations, but also provides some explanation for the apparent long‐term stability of the pools and their clonal structure. Clonal dominance is typically driven through long‐term competition and certainly this is seen in inflationary pools.^83^, ^84^ However, if central memory pools are laid down early,^17^ they will have more diverse TCRs available and so the expanded pools would likely reflect this. Also, clonal stability is observed over very long periods (in human studies),^84^ which seems somewhat at odds with a relatively short half‐life (assuming human and mouse studies are comparable in this respect). However, if cells can both expand (and therefore compete) and maintain themselves in the proliferative pool, this might explain how dominant clones may arise and can survive.

Overall model 2 (a 2‐stage model) seems to be an advance over the simplest models and is backed up by evidence for both stages. There are plenty of unanswered questions, so this concept needs to be challenged experimentally using both the CMV system (where modulations of the memory pool can lead to changes in antigen expression) and in the more antigenically stable and potentially more tractable adenovirus system.

## 6 NEXT STEPS

Recombinant adenoviral and cytomegalovirus vectors are very exciting tools for the priming of human immune responses, with potential applications across a range of diseases including severe acute infections such as Ebola and Respiratory Syncitial Virus (RSV), chronic infections such as hepatitis C virus (HCV), tuberculosis and HIV, and cancers.^27^, ^28^, ^30^, ^31^, ^32^, ^85^ However, we still have much to learn about how T‐cell memory is induced and maintained. Even if it is not called memory “inflation” in all studies, there are clearly sustained functional memory responses induced which include an effector‐memory phenotype and which are maintained long term. These fit well with the slightly updated version of the definition included in this review. For the rhesus macaque‐based cytomegaloviruses, there are a series of additional modifications regarding immune evasion and, interestingly, cellular targeting which can have quite profound impacts on the specificities observed, some of which are unconventional (eg, HLA‐E restricted) and can have potentially potent antiviral properties.^86^, ^87^, ^88^ Overall—even in settings where more conventional CD8^+^ T‐cell responses are induced—there is evidence that these responses can provide protection against viruses and cancers.^2^, ^43^ Thus, understanding what the rules are that govern the induction and maintenance of these populations is very relevant to vaccines and so the models proposed have translational relevance.

The other area where memory inflation is mentioned frequently and where the mechanisms additionally play a role is in aging.^89^ This has been reviewed elsewhere and chiefly in the context of the role of CMV.^20^ There is quite conflicting evidence of the impact of CMV although newer data focusing on subsets of CMV‐seropositive aged populations who have poor control over the virus provide a more convincing association between infection and clinical outcomes.^14^, ^90^, ^91^ Since at a minimum, memory inflation is driven by antigen, a loss of control over virus (as evidenced, for example indirectly via higher IgG levels or directly by detection in monocytes or in urine) should be manifest as enhanced inflation. This interpretation would mean that memory “inflation” (in these cases judged by an exaggerated version of the effector‐memory responses described here) was a co‐correlate or marker of adverse clinical outcomes associated with aging rather than a driver of it, but nevertheless why control over the virus should be lost is not understood and investigation of this may bring us back to the functionality of virus‐specific CD8^+^ T cells. Since the majority of the world's population is infected by CMV, even if the impact of such effects on aging is only part of a bigger picture, they are still likely to be important. There is such a large overlap between the impact of CMV and the effects of aging on immune population structures that this area will continue to attract much attention.

For these 2 reasons—designing better vaccines against major health threats, and defining the role of the immune system in aging—a better definition of the processes underlying memory inflation is a worthy objective. In developing a model which can explain the primary features and the experimental data derived from different groups, there are 3 standout areas which need further work. Firstly, what is the nature of the long‐lived antigen depot and which cells present to the immune system during inflation. The presentation of a subset of peptides is a distinctive feature of inflation indicating a specific pathway is involved and the cross‐correlation of data between different groups and models points to a non‐classical antigen‐presenting cell. Defining what populations are necessary and what is sufficient here for maintenance and priming (and what may differ between viruses) is an important future goal. Directing antigen to express in specific sites is possible, especially with the simplified adenoviral system and could provide an important insight into this process.

Secondly, what are the cells that engage with these populations in tissues. This is a more complex issue in some ways as the definitions vary but the emergence of single cell RNAseq approaches will be hugely valuable here.^92^ This approach also allows some tracking or reconstruction of cellular development which may allow correlation of specific surface markers with specific cellular functions. This approach and similar high content cytometric approaches combined with bioinformatics aimed to address this specific issue will be very valuable in addressing not only the academic questions but also what is achieved through vaccination.

Finally, where is this all happening and what is the structure of memory populations in tissues. A dynamic model with continual replacement still begs questions about the status of long‐lived resident memory cells in tissue, and more deeply where are these sited and what antigen‐presenting cells can they engage with. How such diverse antiviral populations—which share specificity but are diversified among many tissues throughout the body—are related and what their functions are in situ really is a question which underpins all of this work. This can be better addressed with new techniques in intravital imaging and also high content approaches to histochemistry which are emerging rapidly.

Overall, the field of memory inflation has expanded enormously and attracted plenty of attention from different groups studying memory, aging, and host defense. It is still relatively small, however compared to the study of immune exhaustion, a development pathway in the context of chronic infections with which it shares many important features. Perhaps if we can pinpoint the differences in antigen‐presenting cells and in populations responding to antigen (and where)—ie, better define the model proposed—we can at the same time explain why some infections (and cancers) provoke exhaustion rather than inflation and really shed light on this process. This would have a much broader impact still—not only in terms of basic immunology but also for translational studies. Hopefully after another decade and a half, these ambitions will have been realized.

## CONFLICT OF INTEREST

The author has no conflict of interest to declare.
